# The effectiveness of a primary care nursing-led dietary intervention for prediabetes: a mixed methods pilot study

**DOI:** 10.1186/s12875-017-0671-8

**Published:** 2017-12-21

**Authors:** Kirsten J. Coppell, Sally L. Abel, Trish Freer, Andrew Gray, Kiri Sharp, Joanna K. Norton, Terrie Spedding, Lillian Ward, Lisa C. Whitehead

**Affiliations:** 1 Edgar Diabetes and Obesity Research, Department of Medicine, Dunedin School of Medicine, University of Otago, PO Box 56, Dunedin, 9054 New Zealand; 2 Kaupapa Consulting Ltd, Napier, 4110 New Zealand; 3 Health Hawke’s Bay – Te Oranga Hawke’s Bay, PO Box 11141, Hastings, 4158 New Zealand; 4 Department of Preventive and Social Medicine, Dunedin School of Medicine, University of Otago, PO Box 56, Dunedin, 9054 New Zealand; 5 School of Nursing and Midwifery, Edith Cowan University, 270 Joondalup Drive, Joondalup, 6027 Australia

**Keywords:** Prediabetes, Dietary modification, Weight loss, Structured intervention implementation, General practice, Primary care nursing, Indigenous population, Pragmatic clinical trial, Outcome and process assessment, Qualitative evaluation

## Abstract

**Background:**

Primary care nurse-led prediabetes interventions are seldom reported. We examined the implementation and feasibility of a 6-month multilevel primary care nurse-led prediabetes lifestyle intervention compared with current practice in patients with prediabetes, with weight and glycated haemoglobin (HbA1c) as outcomes.

**Methods:**

This study used a convergent mixed methods design involving a 6-month pragmatic non-randomised pilot study with a qualitative process evaluation, and was conducted in two neighbouring provincial cities in New Zealand, with indigenous Māori populations comprising 18.2% and 23.0%, respectively. Participants were non-pregnant adults aged ≤ 70 years with newly diagnosed prediabetes (HbA1c 41-49 mmol/mol), body mass index (BMI) ≥ 25 kg/m^2^ and not prescribed Metformin. A structured dietary intervention tool delivered by primary care nurses with visits at baseline, 2–3 weeks, 3 months and 6 months was implemented in four intervention practices. Four control practices continued to provide usual care. Primary quantitative outcome measures were weight and HbA1c. Linear and quantile regression models were used to compare each outcome between the two groups at follow-up. Qualitative data included: observations of nurse training sessions and steering group meetings; document review; semi-structured interviews with a purposive sample of key informants (*n* = 17) and intervention patients (*n* = 20). Thematic analysis was used.

**Results:**

One hundred fifty-seven patients with prediabetes enrolled (85 intervention, 72 control), 47.8% female and 31.2% Māori. Co-morbidities were common, particularly hypertension (49.7%), dyslipidaemia (40.1%) and gout (15.9%). Baseline and 6 month measures were available for 91% control and 79% intervention participants. After adjustment, the intervention group lost a mean 1.3 kg more than the control group (*p* < 0.001). Mean HbA1c, BMI and waist circumference decreased in the intervention group and increased in the control group, but differences were not statistically significant. Implementation fidelity was high, and it was feasible to implement the intervention in busy general practice settings. The intervention was highly acceptable to both patients and key stakeholders, especially primary care nurses.

**Conclusions:**

Study findings confirm the feasibility and acceptability of primary care nurses providing structured dietary advice to patients with prediabetes in busy general practice settings. The small but potentially beneficial mean weight loss among the intervention group supports further investigation.

**Trial Registration:**

ANZCTR ACTRN12615000806561. Registered 3 August 2015 (Retrospectively registered).

**Electronic supplementary material:**

The online version of this article (10.1186/s12875-017-0671-8) contains supplementary material, which is available to authorized users.

## Background

Diabetes prevalence continues to increase worldwide [1, 2]. Effective prediabetes management strategies to reduce increasing diabetes-related costs, both societal and individual are urgently needed. Amongst New Zealand adults aged ≥15 years the prevalence of diabetes and prediabetes is 7.0% and 25.5%, respectively [3]. Of those with prediabetes, each year an estimated 5–10% develop type 2 diabetes mellitus (T2DM) [4, 5], with most eventually developing the condition, particularly those who are overweight or obese [6].

In New Zealand screening for diabetes and prediabetes is recommended as part of cardiovascular risk assessment for men aged ≥45 years and women aged ≥55 years, and at younger ages for high risk groups, including indigenous Māori, Pacific, Indo-Asian, obese, those with a family history of diabetes and women with previous gestational diabetes [7]. It is assumed that general practitioners (GPs) and primary care nurses will deliver effective nutrition advice for the management of prediabetes and prevention of diabetes. However, although good clinical trial evidence demonstrates lifestyle advice prevents progression from prediabetes to T2DM [8, 9], similar results have not been demonstrated in ‘real-world’ general practice settings [10], partly because few studies have examined the translation of diabetes prevention clinical trial evidence into the primary care setting [11].

Of the few primary care-based diabetes prevention lifestyle interventions, most have utilised GPs [12], dietitians [13] or multidisciplinary teams with nutritionists, exercise specialists and lifestyle coaches [14–17], which are costly. Yet approaches to cholesterol-lowering dietary advice in the general practice setting in the 1980s and 1990s and a recent general practice-based weight loss study suggest it is better if a nurse takes the lead for lifestyle advice with the GP in a supportive role [18, 19].

This paper reports on the results of the Prediabetes Intervention Package (PIP) in primary care pilot study which aimed to examine the implementation and feasibility of a multilevel primary care nurse-led prediabetes lifestyle intervention compared with current practice on weight and glycated haemoglobin in patients with prediabetes, at 6 months.

## Methods

This primary care-based prediabetes intervention study used a convergent mixed methods design [20], combining a 6-month pragmatic non-randomised quantitative pilot study [ACTRN12615000806561] with a qualitative process evaluation to assess intervention implementation.

### Setting

The study was conducted in general practices and community settings in two neighbouring provincial cities in New Zealand with populations of 57,240 (18.2% Māori) and 73,245 (23.0% Māori) in 2013 [21]. Māori, the indigenous population of New Zealand, have high rates of prediabetes (30.4%) and diabetes (9.8%) [3].

In New Zealand primary medical care is delivered by GPs in mostly group, but some solo practices. Almost all practices have government capitation funding with varying levels of patient co-payment, depending on age and socioeconomic status of patients and the practice’s business model. Most GP practices employ primary care nurses and belong to a Primary Health Organisation (PHO). PHOs are not-for-profit organisations that provide primary health services either directly or through their provider members. There was a single PHO in the study region.

For this study, member general practices employing primary care nurses were recruited. For operational reasons and to minimise potential contamination between the two arms of the trial, four intervention practices were located in one city and four control practices in the other. Practice patient characteristics were similar between arms, and included those with a high proportion of Māori and those with a high deprivation index score [22].

### Participants and recruitment

Eligible participants were non-pregnant adults aged ≤70 years with newly diagnosed prediabetes (HbA1c 41-49 mmol/mol or fasting plasma glucose 6.1–6.9 mmol/L) [7], a body mass index (BMI) above 25 kg/m^2^, not prescribed Metformin and able to communicate in English. Newly diagnosed prediabetes meant a diagnosis within the previous 6 months and no documented appointment to discuss prediabetes management following a positive test. Diagnosis followed either screening due to identified risk of prediabetes or cardiovascular risk assessment [7].

Practice patient management systems identified and generated a list of existing eligible patients. Eligible patients received a study invitation letter from their practice and a follow-up phone call from the primary care nurse. An appointment was arranged with those agreeing to participate. Patients subsequently diagnosed with prediabetes who fulfilled the eligibility criteria were invited to participate in the study at time of diagnosis. Patient recruitment occurred between August 2014 and April 2015.

### Intervention

The intervention was informed by a literature review of lifestyle interventions and designed in collaboration with the PHO. The focus was to provide patients and their family/whānau^1^ with an understanding of healthy eating principles and enhance empowerment around dietary choices. The multilevel package comprised six components:

1. Health professional training and support - evidence-based culturally appropriate training package for primary care and community nurses, with dietitian support.

The intervention primary care and community education nurses participated in a 6-h theoretical and practical training course, which included nutrition principles, dietary assessment, goal setting, the context within which nutrition advice is given and how to measure height, weight and waist circumference. Course dietary content was based on successful translation of diabetes treatment and prevention guidelines into a clinical trial setting in the Lifestyle Over and Above Drugs in Diabetes (LOADD) study [23]. Primary care dietary assessment, internal and external factors affecting food choices, cultural influences on diet, behaviour change and effective communication of dietary information were included in the course, which was delivered by study investigators (KC, KS and JN) with input from a local dietitian. A training manual provided reference material and research protocols.

A follow-up 2-h session was held to review study protocols, answer questions and make any necessary practical changes to the protocol. A further 2-h update course was run at 6 months, using case studies delivered by intervention primary care nurses to illustrate particular dietary consultation challenges.

A dietitian arranged monthly case review meetings with the primary care nurses. Similarly, a liaison nurse (TS) visited participating nurses at least monthly to assess intervention adherence and provide advice. Dietitian and nurse visits alternated, and both were available by email and phone to answer questions or discuss specific cases.

2. Individual patient education - dietary assessment, goal setting and dietary advice sessions

After providing written consent, patient participants were offered an initial 30 min dietary session with the primary care nurse and encouraged to bring family/whānau. Immediately prior to their nurse appointment, they completed a brief dietary assessment. We used Starting the Conversation (STC):Diet, a validated eight-item simplified food frequency instrument designed for use in primary care and health-promotion settings [24]. STC:Diet was minimally modified, with permission, for the New Zealand context. We changed the word ‘sodas’ to ‘soft drinks’, and added a traffic light system to indicate healthy, not-so-healthy and unhealthy dietary habits. The nurse reviewed the STC:Diet responses; asked additional dietary prompt questions (developed by KS and JN); sought additional contextual information, such as household membership and budget, who purchased household foods and specific dietary requirements/ choices such as vegetarianism; and took anthropometric measures (height, weight and waist circumference). We called these additional questions the Detailed Dietary Assessment (DDA). A weight goal of a 5–10% loss over 6 months was calculated. Responses to the STC:Diet and DDA informed three dietary goals, negotiated with the participant, and individualised tailored dietary advice. Funded 15 min follow-up appointments were arranged 3 weeks later, then at 3 and 6 months.

3. Key messages and consistent opportunistic reminders

The three dietary goals were recorded in the practice patient management system for each participant. This facilitated opportunistic targeted advice and guidance by participants’ GPs, thus reinforcing dietary information provided by the nurses. The goals were reviewed and updated accordingly, at follow-up nurse appointments.

4. Nutritionally supportive primary care environment

Prior to study beginning, each intervention practice was visited to discuss ways to enhance dietary messages provided by nurses. Specifically, the dietary information provided in pamphlets, magazines and posters in the waiting rooms were reviewed and updated, so dietary messages were appropriate and consistent. Provision of magazines that supported reputable dietary messages and active living, hobbies and sports, and posters promoting fruit and vegetables, such as those offered by Vegetables.co.nz (www.vegetables.co.nz), were encouraged.

5. Community-based group education for patients and their family/whānau

Community group nutrition education courses consisted of six weekly sessions of 1–1.5 h each. Courses were delivered by community nurses from the local Sports Trust at various times and locations and aimed to provide generic nutrition knowledge and advice. Content was developed by the research dietitian (KS), in conjuction with Sports Trust personnel. Topics included prevention of progression to diabetes, food groups, label reading, eating out, menu planning and food safety.

6. Written Patient Resources

Readily available patient resources were utilised. The key resource was the Diabetes New Zealand booklet, *Diabetes and healthy food choices* [25], used successfully in the LOADD study [23], where participants found the information clearly presented and easily understood.

### Control practices (usual care)

Primary care nurses at control practices continued to provide dietary advice to patients with prediabetes in their usual way. Usual care is based on the Prediabetes Advice guidance circulated to all general practices by the New Zealand Ministry of Health in August 2013 [26]. Lifestyle advice is based on goal setting, a weight loss of 0.5-1 kg per week and a long term loss of at least 5% in those who are overweight or obese, healthy eating, aiming for 30 min of moderate intensity exercise most days, regular follow-up and a repeat HbA1c test following 3 months of ‘lifestyle therapy’, then 6–12 monthly. This typically consists of unstructured advice and sometimes a ‘green prescription’ [27], which is a nationwide initiative designed to increase physical activity (http://www.health.govt.nz/our-work/preventative-health-wellness/physical-activity/green-prescriptions).

### Physical activity - intervention and control practices

All participating patients were given standard advice on physical activity, that is, 30 min of physical activity of moderate intensity on most, if not all, days of the week. Each participant was also given a ‘*Be Active Every Day’* pamphlet [28].

### Quantitative data

The primary outcome measures were weight and HbA1c. Other outcome measures included waist circumference, body mass index, blood pressure and lipids. The patient management system was modified to facilitate the recording of study data at baseline and 6 months. Most data were collected as part of routine primary care practice, and included demographic and medical details, lifestyle information (smoking, alcohol, diet and physical activity), blood pressure, anthropometric measures (height, weight and waist circumference) and laboratory measures (HbA1c, glucose, lipids, urate and liver enzymes). Additional non-routine intervention data included dietary assessment, dietary goals and weight goal.

Participating nurses were trained on standard practices for measuring anthropometry and blood pressure. General practice stadiometers, weighing scales and sphygmomanometers were calibrated. A Lufkin Executive thinline (2 m) tape measure was provided to each practice for measuring waist circumference. Shoes were removed before conducting anthropometric measurements. Weight was measured with patients wearing one layer of light clothing and waist circumference was measured with the tape measure against the skin. Nurses were asked to take duplicate measures.

#### Sample size calculations

A standard deviation of 18 kg for weight and a correlation between baseline and follow-up weights of 0.95 were obtained using data from the 2008/09 New Zealand Adult Nutrition Survey [29] and 2005 data from the Otago Diabetes Register [30], retrospectively. To detect a difference of 4 kg in weight (equivalent to a 5% relative difference in weight loss for a patient initially weighing 80 kg) at 6 months with 90% power using a two-sided test at the 5% level, required 42 people in each group with complete data. After incorporating design effects based on a mean cluster size of 21 (based on 4 practices in each arm) and an intraclass correlation (ICC) of 0.03, and allowing for a 20% loss to follow-up, required 84 people in each arm of the study at baseline.

#### Statistical analysis

Demographic and health characteristics were compared between the two groups using Chi-squared and Fisher’s Exact tests at baseline and follow-up, without adjusting for practice cluster effects. Similarly retention at 6 months was compared within each group. Linear regression models were used to compare each outcome between the two groups at follow-up, except for GGT for which quantile regression was used due to extreme skew. Each outcome was adjusted for baseline values, sex, alcohol consumption, family history of T2DM, and ethnicity. The number of clusters was small, and Huber-White robust standard errors were used with Froot’s extension for clustering at the practice level. Bias corrected confidence intervals were obtained from 1000 bootstrapped samples for each outcome (random number seeds were specified for each outcome in the statistical analysis plan prior to all analyses). Standard model diagnostics were used including assessing residual normality and homoscedasticity using histograms and scatterplots of residuals. Log-transformations were investigated and used where this improved residual diagnostics. Multinomial logistic regression was used to compare weight change categories (≥ 5% weight loss; < 5% weight loss; no weight change or weight gain) with clustered robust standard errors. As this was a pilot study no formal adjustments were made for multiple comparisons. Stata 14.2 was used for all analyses with two-sided *p* < 0.05 considered statistically significant.

### Process evaluation

A summative process evaluation using qualitative research methods was undertaken to explore, whether the prediabetes intervention was implemented as intended (intervention fidelity), the feasibility of implementing the intervention package in busy primary care settings and what aspects of intervention implementation worked well and what was challenging from both stakeholder and patient perspectives. Data were collected through three pathways: Observation of nurse training sessions and monthly steering group meetings with comprehensive note taking, document review, and interviews with key informants (*n* = 17) and intervention patients (*n* = 20). Data were collected by SA, an independent health researcher with 20 years qualitative research experience in multi-ethnic communities, and who was not involved in the intervention design and implementation.

All interviewees gave prior written consent and all agreed to audio-taping. Interviews were semi-structured with open-ended questions (Additional file 1).

Key informants were purposefully selected [31] as key players in intervention implementation, and included all intervention primary care nurses (*n* = 11). All those approached agreed to participate. Interviews were undertaken at the workplace or another chosen venue between 30 June and 21 August 2015. Primary care nurses who worked together were interviewed in pairs. They were asked about their intervention role and experiences, perceptions of successes and challenges, and recommendations for future development. Comprehensive notes were taken at the interview and after re-listening to audio-recordings. Preliminary findings from analysis of these interviews were presented to participants as a group and feedback encouraged. Written notes from this session were included in the final dataset.

Patients who had completed the six-month intervention were purposefully selected to ensure a range of demographic profiles and glycaemic outcomes (Table 1). They were first approached by their primary care nurse and, if willing to participate, contact details were given to SA. Four declined; two were too busy and two gave no reason. SA phoned those wishing to participate to explain the research and arrange an interview. All were interviewed individually between 7 August and 15 December 2015 at their chosen location; their own home (*n* = 17) or the researcher’s workplace (*n* = 3). Although they had the option of including a support person, none did. Interviews explored patients’ experiences of the intervention, both enjoyable and challenging. Close attention was paid to cultural etiquette when interviewing Māori and Pacific patients. Total interview time was 45–60 min. At interview conclusion, patients were given a $NZ20 gift voucher in appreciation of their time. All patient interview audio-recordings were transcribed by an external transcriber who had signed a confidentiality agreement. Transcripts were read thoroughly by SA to check for accuracy. The data were anonymised and password protected.

**Table 1:** Demographic characteristics and glycaemic outcomes for the 20 intervention participants who were interviewed

Patient participants	Normoglycaemia	Prediabetes	Diabetes	Total
Māori female		4		4
Māori male	1	3	1	5
NZ European female	2	2	1	5
NZ European male	1	3		4
Pacific female		1		1
Pacific male		1		1
Total	4	14	2	20

### Qualitative data analysis

The data were analysed using thematic analysis [32]. SA, LW and KC undertook multiple close readings of the transcripts. Data coding and initial theme development were undertaken by SA, reviewed by LW and KC and discussed together over the course of several meetings. Themes were derived inductively. Key informant and patient interview data were initially coded and analysed separately, then combined, synthesised and final key themes and sub-themes agreed by these three researchers.

## Results

### Characteristics of patient participants

**Fig. 1:**
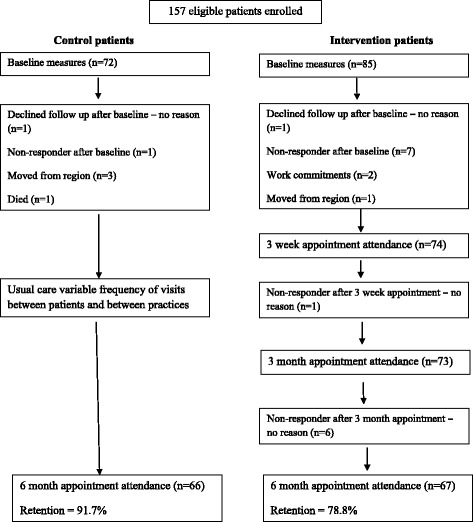
Flow of study participants

Figure 1 shows the flow of the 157 participants enrolled in the study. Baseline characteristics and retention rates are shown in Table 2. At baseline there were slightly more men, and almost one-third were Māori. A family history of T2DM was common (39.5%), as were diabetes-associated co-morbidities - hypertension (49.7%), dyslipidaemia (40.1%), gout (15.9%), ischaemic heart disease (9.6%) and stroke (4.4%). Among women, 5.4% had previous gestational diabetes. Baseline and 6 month measures were available for 91% control participants and 79% intervention participants.

**Table 2:** Demographic characteristics and diabetes-related co-morbidities of participants at baseline and 6-months. Data presented are number (%)

		Control				Intervention				Between groups *p*-values	
		At baseline (*n* = 72)	With follow-up data (*n* = 66)	No 6-month data (*n* = 6)	Retention *p*-value^a^	At baseline (*n* = 85)	With follow-up data (*n* = 67)	No 6-month data (*n* = 18)	Retention *p*-value^a^	At baseline^a^	At 6-months^a^
		*n* (%)	*n* (%)	*n* (%)		*n* (%)	*n* (%)	*n* (%)			
*Age categories (years)*											
	≤ 49	11 (15)	11 (17)	0 (0)	0.719	13 (15)	8 (12)	5 (28)	0.226	0.724	0.582
	50–64	40 (56)	36 (55)	4 (67)		42 (49)	35 (52)	7 (39)			
	≥ 65	21 (29)	19 (29)	2 (33)		30 (35)	24 (36)	6 (33)			
*Sex*											
	Female	28 (39)	25 (38)	3 (50)	0.672	46 (54)	38 (57)	8 (44)	0.429	0.077	0.370
	Male	44 (61)	41 (62)	3 (50)		39 (46)	29 (43)	10 (56)			
*Ethnicity*											
	Māori	22 (31)	19 (29)	3 (50)	0.471	27 (32)	19 (28)	8 (44)	0.001	0.456	0.942
	NZ European & Other	48 (67)	45 (68)	3 (50)		52 (61)	47 (70)	5 (28)			
	Pacific	2 (3)	2 (3)	0 (0)		6 (7)	1 (1)	5 (28)			
*Family history of T2DM*											
	Yes	28 (40)	27 (42)	1 (17)	0.390	34 (40)	24 (36)	10 (56)	0.179	1.000	0.590
	No	42 (60)	37 (58)	5 (83)		50 (60)	42 (64)	8 (44)			
*Alcohol consumption*											
	Above guidelines	8 (13)	8 (14)	0 (0)		6 (8)	5 (9)	1 (6)	1.000	0.573	0.560
	Within guidelines	54 (87)	50 (86)	4 (100)		65 (92)	49 (91)	16 (94)			
*Smoking*											
	Current	12 (17)	9 (14)	3 (50)	0.147	18 (21)	12 (18)	6 (33)	0.340	0.193	0.336
	Never	24 (33)	22 (33)	2 (33)		26 (31)	20 (30)	6 (33)			
	Past – quit >12 months	31 (43)	30 (45)	1 (17)		27 (32)	24 (36)	3 (17)			
	Past – quit <12 months	5 (7)	5 (8)	0 (0)		14 (16)	11 (16)	3 (17)			
*Co-morbidities*											
Hypertension											
	Yes	39 (55)	36 (55)	3 (50)	1.000	38 (45)	30 (45)	8 (44)	1.000	0.260	0.296
	No	32 (45)	29 (45)	3 (50)		47 (55)	37 (55)	10 (56)			
Ischaemic heart disease											
	Yes	7 (11)	5 (8)	2 (40)	0.086	7 (8)	5 (7)	2 (11)	0.639	0.778	1.000
	No	58 (89)	55 (92)	3 (60)		77 (92)	61 (91)	16 (89)			
Stroke											
	Yes	2 (3)	1 (2)	1 (25)	0.122	5 (6)	4 (6)	1 (6)	1.000	0.699	0.367
	No	62 (97)	59 (98)	3 (75)		78 (94)	61 (94)	17 (94)			
NAFLD											
	Yes	4 (7)	4 (7)	0 (0)	1.000	1 (1)	1 (2)	0 (0)	1.000	0.166	0.192
	No	57 (93)	53 (93)	4 (100)		79 (99)	61 (98)	18 (100)			
Gout											
	Yes	13 (20)	13 (22)	0 (0)	0.574	12 (14)	8 (12)	4 (22)	0.271	0.378	0.158
	No	51 (80)	47 (78)	4 (100)		73 (86)	59 (88)	14 (78)			

^a^Chi-squared test where at least 80% of cells have expected counts 5 or above, Fisher’s Exact test otherwise

### Clinical outcomes

Table 3 shows the clinical outcomes at baseline and 6 months. Overall, the control group gained weight (0.8 kg), whereas mean weight for the intervention group decreased (1.3 kg), a 2.2 kg difference. After adjustment for baseline measures the intervention group lost a mean 1.3 kg more than the control (*p* < 0.001). Among the intervention group, 65% lost some weight, and 18% achieved at least a 5% weight loss compared with 32% and 5%, respectively, of those in the control group (unadjusted multinomial regression Wald *p* < 0.001). Small decreases in both HbA1c and waist circumference were observed in the intervention group, compared with small increases in the control group. These differences were not statistically significant. At 6 months four intervention participants and eight control participants had progressed to T2DM.

**Table 3:** Clinical and laboratory measures for participants at baseline and 6 months. Data presented are arithmetic means (SDs) number (%), unless otherwise stated

	Control (*n* = 66)		Intervention (*n* = 67)		Difference in mean changes in intervention group	
	Baseline Mean (SD)	6 months Mean (SD)	Baseline Mean (SD)	6 months Mean (SD)	Ratio (95% CI)	*p*-value
HbA1c (mmol/mol)	43.0 (2.2)	43.8 (6.2)	43.2 (2.2)	42.0 (3.6)	0.96 (0.92, 1.00)	0.096
Weight (kg)^a^	93.7 (15.1)	94.6 (15.5)	96.9 (21.5)	95.6 (23.8)	0.97 (0.95, 0.98)	< 0.001
BMI (kg/m^2^)^b^	33.0 (6.0)	33.3 (6.1)	35.1 (7.4)	34.5 (8.1)	–	
Waist circumference (cm)	104.0 (17.9)	106.0 (12.2)	109.4 (15.2)	107.7 (16.3)	0.97 (0.94, 1.00)	0.101
Systolic blood pressure (mmHg)	132.7 (17.0)	135.0 (16.8)	133.0 (13.7)	131.2 (13.8)	0.97 (0.92, 1.05)	0.422
Diastolic blood pressure (mmHg)	81.2 (10.9)	80.3 (10.7)	79.6 (9.1)	79.2 (9.2)	1.00 (0.94, 1.04)	0.949
Total cholesterol (mmol/l)	5.3 (1.3)	5.2 (1.3)	5.3 (1.4)	5.2 (1.1)	1.00 (0.95 1.08)	0.933
HDL-cholesterol (mmol/l)	1.2 (0.3)	1.2 (0.4)	1.3 (0.5)	1.3 (0.5)	1.02 (0.96, 1.09)	0.595
Triglycerides (mmol/l)	2.4 (1.8)	2.4 (1.7)	2.1 (1.1)	2.2 (1.3)	0.95 (0.79, 1.24)	0.596
Alanine transaminase (IU/L)	33.7 (20.3)	33.4 (18.2)	28.5 (15.2)	26.0 (12.3)	0.91 (0.75, 1.03)	0.206
Aspartate aminotransferase (IU/L)	25.6 (10.3)	25.6 (11.3)	23.9 (7.5)	22.9 (9.7)	0.95 (0.73, 1.12)	0.649
Gamma glutamyl transpeptidase (IU/L)^c^	39.0 (31.5)	41.0 (33.0)	32.0 (61.0)	31.0 (53.0)	−4.00 (−8.19, 2.22)	0.369
Urate (mmol/l)	0.39 (0.11)	0.37 (0.09)	0.36 (0.07)	0.37 (0.08)	0.02 (−0.01, 0.25)	0.799

Statistical comparisons are from linear regression models, except for GGT where a quantile regression model was used to model medians, adjusting for baseline values, sex, alcohol consumption, family history of T2DM, and ethnicity and adjusting standard errors for clustering within practices

^a^6-month weight measurement was missing for one intervention participant. ^b^Ratio not analysed as weight was the preferred body mass outcome. ^c^Log-transformed

### Implementation fidelity

Implementation fidelity was high. All intervention primary care nurses attended the training and update sessions. They delivered the brief dietary assessment, goal setting and appropriate dietary advice, as per the study protocol. This was confirmed, during key informant interviews, by the support dietitian and liaison nurse, who independently checked in with each practice monthly to offer support to the nurses and ensure protocol compliance. The intervention practices reinforced healthy eating messages with appropriate seating, magazines in the waiting room and posters. Five separate group education courses were offered at a range of community settings and times to facilitate uptake and were delivered as planned, as confirmed by the group educators’ manager. Eleven of the 20 patients interviewed visited their GP practice for other health issues during the intervention, eight of whom reported and greatly appreciated their GP providing additional encouragement.

### Intervention feasibility

The process evaluation confirmed the feasibility of implementing the intervention in busy general practice settings. Primary care nurses reported that training ran smoothly, as did intervention implementation. They successfully recruited and mostly retained patients with prediabetes in the programme, and successfully incorporated the intervention into their busy workload, despite experiencing some time and study administrative pressures. The timing of the start of intervention implementation, when nurses needed sufficient time to familiarise themselves with this new additional role, was important to avoid clashes with increased seasonal-related workloads, such as flu vaccinations. Timely patient follow-up was affected when appointments fell in and around the festive season and patients were away or busy. GPs opportunistically encouraged their participating patients, utilising the information, including established dietary goals, recorded on the patient management system.

The community education component was also feasible, with group educators being very committed and adjusting usual service delivery processes to contribute to the study. Uptake was less than optimal, with 53% of intervention patients attending any sessions and one-third of these not completing the course. Modifications were recommended, including that six sessions be reduced to three or four.

### Intervention acceptability

Key informants and patients alike found the intervention to be highly acceptable. It was described as “*well thought out and well planned as an initiative*” (KI-13, primary care nurse) and “*a very positive experience*” (KI-1, liaison nurse). One Māori woman recommended it “*roll out to maraes* 2 *and a lot of the community groups*” (Pt-15) alluding to the important need for improvements in dietary options at traditional meeting places and community events.

Five sub-themes relating to implementation acceptability and success were identified; strong relationships, primary care nurse empowerment, simplicity of approach, clear information and resources, and group support. Findings were consistent among primary care nurses working with differing communities and among patients with differing demographic and glycaemic outcome profiles.

1. Strong relationships

A major factor contributing to intervention acceptability and success was strong relationships between all parties. The smooth implementation process was facilitated by good communication between the different stakeholder groups and a shared desire to address a significant health issue. Monthly steering group meetings involving stakeholder group representatives enabled issues to be discussed and addressed as they arose. The liaison nurse, a local specialist diabetes nurse, knew many primary care nurses and these pre-existing relationships appeared to facilitate her role.

Most patients expressed their strong appreciation of the opportunity to proactively address their recently diagnosed prediabetes and saw the care and attention provided by their primary care nurse and group educator and their enhanced relationship with them as a powerful enabler:

*“It was the way she encouraged me, how she uplifted me. I am so grateful... So I think having the right people at the forefront there just to open you up, you know, and acknowledging where I am at.”* (Pt-8, Pacific woman).

*“Just by talking with them it makes you want to motivate yourself, you know. And you realise that they’re not doing it for them, they’re doing it for you. And to have that support that you don’t know is out there, that’s brilliant, that’s absolutely brilliant”* (Pt-9, Māori man).

2. Nurse empowerment

Primary care nurses felt the information, strategies and structured approach, along with nurse and dietitian expert advice and support, equipped them well to provide dietary advice to their patients with prediabetes. They felt newly empowered to work more effectively and intensively with these patients *“Spending time with them and giving them the education. I found that really rewarding”* (KI-15 primary care nurse).

3. Simple approach

Primary care nurses and patients both praised the intervention’s simplicity. The nurses found focusing on small manageable goals both practical and realistic.

*“I was quite happy to say to people ‘what we’re going to do, the changes, it’s all simple…..and I think they went away not thinking it was a humongous ask on the food changes.”* (KI-12, primary care nurse).

Patients also appreciated focussing on simple, achievable, individually tailored dietary goals which they felt made making dietary improvement entirely manageable.

*“It wasn’t stop this, stop that. It was cut down on this, cut down, little steps... The favourite saying is ‘little steps’. And that’s probably one of the most helpful sayings I’ve ever heard.”* (Pt-9, Māori man).

*“That (setting achievable goals) was explained and there was a fair bit of time put into that... You know, especially around Māori or Polynesian people, food can be a blessing and not a blessing. (Laughter). But it’s certainly hard to change things that you’ve done all your life. And I think the nurses that I had anyway were very helpful and supportive...[they] had good ideas.”* (Pt-13, Māori man).

4. Clear information and resources

The clarity and consistency of information and resources also contributed to intervention acceptability. This was significant for many patients as they grappled with the implications of their diagnosis. One reported finally gaining clarity after being confused by the plethora of dietary information received when supporting her husband who had had diabetes.

*There’s so much out there now that just totally throws you every which way and you don’t know what’s right and what’s wrong. And it took away some of those falsehoods that were out there... It was easy to follow, it was easy to understand. The complication was taken out of it.* (Pt-1, European woman).

*They gave us all the resources to say you have options in how you want to change your lifestyle... That’s what I took out of it, is that the information was readily available and the guidance was there, and the help. I have nothing but praise for all parties involved.* (Pt-11, Pacific man)

5. Group support

Although six of the 20 interviewed patients reported being uncomfortable in groups and did not attend or did not complete a group education course, the rest enjoyed being with people who were “*in the same boat*”. They liked hearing other people’s stories, sharing their own experiences with an interested audience, exchanging ideas and strategies, and being motivated by others. For some, this was another facet of support enabled by the intervention.

*“The good thing was it brought you in contact with other people in your situation. That’s a major, that’s a good thing, you know”* (Pt-2, Māori man).

### Challenges

A number of challenges to implementing the intervention were identified. These were described primarily by key informants, as many patients spoke only positively about the implementation process, irrespective of their weight or glycaemic outcome. All patients, however, identified challenges they encountered when making dietary changes in answer to a separate question, which will be reported elsewhere. The main challenge identified by key informants was the need for greater information exchange between the primary care and community group educator teams. Neither group appeared to have a full understanding of what the other offered. While both groups had been present at the training sessions, at that time the group education courses had not been finalised. Key informants felt strengthening this linkage could enhance intervention cohesion and possibly group education attendance.

There was remarkable consistency between key informant and patient feedback on other implementation challenges. While primary care nurses did accommodate intervention sessions into their busy schedules, some nurses and patients felt more time was needed, particularly during the initial appointment when study procedures had to be complete. Both nurses and patients also suggested that the 3-month gap between the third and fourth appointments was too long, possibly leading to patients losing motivation. More sessions or monthly phone check-ins were recommended.

Developing goals that were realistic and manageable for patients with low food budgets was a significant challenge for primary care nurses. Some nurses called on the dietitian’s expertise in these cases and developed effective pragmatic strategies but this was an ongoing challenge for patients with very low budgets. A few patients also identified this as a real challenge. One man, who had been made redundant and struggled financially, repeated several times that this was his biggest barrier and cautioned health professionals not to put unrealistic dietary expectations on people with limited financial resource:

*“Look, the barrier to those goal settings is budget, you know... So when you see on TV people saying they’re eating unhealthily, what they’re doing, what we’re doing is we’re eating to a budget planned to survive for the week.... So don’t go telling poor people, you’re going to get diabetes if you eat this and this and this, so we want you to eat this food, but it’s too expensive for you to buy, you know.”* (Pt-2, Māori man).

## Discussion

Our structured prediabetes dietary intervention was able to be competently delivered by primary care nurses within the busy general practice setting following a 6-h training session, a 2-h case study nutrition update and monthly dietitian support for up to 9 months. We found this primary care nurse-delivered intervention led to twice as many intervention participants losing weight at 6 months compared with control participants. Overall the intervention group lost 1.3 kg while the control group gained 0.8 kg, a 2.2 kg difference between the groups, and 18% of intervention participants lost at least 5% of their baseline weight compared with 5% of control participants (*p* < 0.001). HbA1c decreased in the intervention group and increased in the control group. While there were clinically significant changes for some individuals, the difference between the two groups was small and not statistically significant. This study was not powered to examine progression to T2DM, but promisingly the number of intervention participants who progressed to T2DM at 6 months (*n* = 4) was half that of control participants (*n* = 8).

The less than expected mean weight loss and insignificant change in HbA1c may reflect insufficient intensity of the intervention as both nurses and patients recommended additional sessions or monthly phone check-ins, particularly between months 3 and 6 of the intervention. This is a highly likely explanation considering that during the first 6 months of the DPP lifestyle intervention, case managers met with individual participants at least 16 times during the first 24 weeks of the study, [33] compared with only 4 appointments during our 6 month intervention. However, although mean weight loss was relatively small in our study, it is clinically meaningful, as in the DPP for each kilogram of weight loss, the risk of progressing to diabetes was reduced by 16% [34].

Effective evidence-based management of prediabetes in the primary care setting is potentially a key strategy to help stem the diabetes epidemic worldwide. This is an international challenge [35]. Primary care nurses are ideally suited to lead lifestyle changes, often building on an already established relationship, but for many nurses appropriate nutrition knowledge and skills, and resources are lacking [36]. The results from our feasibility study are encouraging when compared with the relatively few studies where primary care nurses have been upskilled and trained to deliver a prediabetes intervention programme. An evaluation of the Dutch Diabetes Federation *‘Road map towards diabetes prevention’* one-year nurse-led intervention found that while the level of reported physical activity increased in the intervention group compared with the control group, there was no difference in BMI at 2 years between the groups [37]. In contrast, among 105 participants in the Polish arm of DE-PLAN, a European-wide primary healthcare intervention based on the principles of the Diabetes Prevention Study [9], weight significantly decreased by 2.27 kg (*p* < 0.001) at 1 year [38] but increased by 1.14 kg at 3 years. While our 79% attendance at all four programme primary care nurse visits was less than ideal, it appears to be as good as [39] or better than other programmes [13, 37, 38]. Further, of six intervention participants who attended the 3-month but not 6-month visit, one achieved normoglycaemia at 3 months, and may have deemed it unnecessary to continue in the programme. For three others, HbA1c declined noticeably for two, and remained the same for another, suggesting they too had gained some benefit from two or three nurse-led intervention visits.

The qualitative process evaluation found that extending primary care nurses’ dietary knowledge and practice base, and incorporating this into the everyday work of primary care, was not only feasible but also effective and rewarding. Further, the intervention was implemented as intended and highly acceptable to both nurses and patients. A central theme was the importance of strong cooperative relationships at all levels for effective, successful intervention implementation. A significant enabler was good communication and relationships between the funding PHO and primary care practices, between the primary care nurses and their liaison nurse and dietitian, and between the patients and their nurses and groups educators. The pre-existing and ongoing relationship between the nurse and patient was portrayed as one of trust and respect, and appeared to be an important underpinning success factor. The importance of effective communication when providing lifestyle advice and support has been increasingly recognised. Good relationships with primary care professionals were identified as an important component of dietary advice and support among those seeking treatment for obesity [40]. Conversely, Ball et al. [41] identified the lack of an established and ongoing relationship with a dietitian, and advice that was too directive and not individualised as key negative issues in a group of patients recently diagnosed with T2DM receiving nutrition advice from a dietitian. These findings are consistent with those of Ciechanowski et al. [42] who found that poor communication between healthcare providers and patients with diabetes may have a negative effect on treatment adherence. Indeed, in a small qualitative study, women with diabetes rated patient-provider communication as the most important factor influencing their adherence to diabetes treatment [43].

The significant influence of cultural and socioeconomic factors on diet is well documented [44–46]. Our intervention was not prescriptive, but facilitated a structured approach taking into account individuals’ different socioeconomic and cultural environments, which enabled nurses to work with patients to first readily identify less than ideal dietary patterns, then develop individualised achievable goals and activities to improve their dietary practices. This approach appeared to work well for the Māori and Pacific participants interviewed. A recent study exploring perspectives on dietary diabetes education and healthy food choices among Pakistani people with T2DM [47] similarly concluded that dietary education that aims at establishing a connection to the everyday life of patients can facilitate successful and sustainable changes in dietary practices.

Recommendations from the process evaluation to further improve the intervention implementation process and its reach included increasing the patient’s primary care nurse sessions from four to six; decreasing the group education sessions from six to four and ensuring good information flow between primary care nurses and community educators. These have been incorporated into the subsequent implementation of the intervention in further general practices in the study region.

Important considerations underpinned our study design. We used a convergent mixed methods design to assess the intervention [20] to take into account the pragmatic real-world setting and the principles of implementation science [48]. In real-world settings external factors that cannot be controlled may influence intervention implementation and effect, and this was a key reason for including a qualitative process evaluation. Qualitative evaluations of interventions are seldom reported but, as we found, can provide valuable insight into the intervention process, and the feasibility and acceptability of interventions [49–51].

Key strengths of this study were the ability of practices to fully embed the intervention within usual care, and the full engagement of primary care nurses at both intervention and control practices. This allowed the intervention to be adequately assessed, and facilitated improvements, a necessary step in the development and testing of general practice delivered lifestyle intervention for patients with diabetes [52, 53]. A high level of participation among Māori was also an important and critical strength, given the high prevalence of diabetes among this indigenous group [3]. A limitation was that primary care nurses, who first approached potential patient participants for the process evaluation, may have been more likely to choose those with whom they had good relationships. However, there were no other avenues for researchers to approach patients. Also, as we did not interview GPs, our assessment of their involvement was ascertained indirectly via participating nurses and patients. This study was a pragmatic non-randomised feasibility study, and the effectiveness of the intervention cannot yet be confirmed. While it is likely that weight loss among the intervention group was due to our primary care nurse-led dietary intervention, an alternative explanation is regression to the mean. Baseline weight measures differed between the two groups (93.7 kg and 96.9 kg), and at 6 months the mean weight for the intervention group (95.6 kg) was still greater than the control group at either time.

## Conclusions

Study findings confirm the feasibility and acceptability of primary care nurses providing structured dietary advice to patients with prediabetes in busy primary care practices. Consideration of socioeconomic and cultural factors enabled realistic achievable nutrition goals to be established. Although this was a 6-month pragmatic pilot study, improvements in anthropometric measures and the positive trusting relationships between patients and primary care nurses suggest this programme is a worthwhile potentially long term primary care-based diabetes prevention intervention. Increased intensity of the intervention may be necessary to achieve greater weight loss, and definitive randomised controlled trials are required to assess intervention effectiveness.

## Additional file

Additional file 1: Prediabetes Intervention Package (PIP) in primary care study process evaluation - key informant and intervention patient interview guides. This file contains copies of the process evaluation interview guide for key informants and the interview guide for intervention participants. (PDF 89 kb)

## Abbreviations

BMI: Body Mass Index
DDA: Detailed Dietary Assessment
GP: General Practitioner
HbA1c: Glycated haemoglobin
ICC: Intraclass Correlation
LOADD: Lifestyle Over and Above Drugs in Diabetes study
PHO: Primary Health Organisation
PIP: Prediabetes Intervention Package in primary care
STC:Diet: Starting the Conversation:Diet
T2DM: Type 2 Diabetes Mellitus

## Footnotes

1 Māori language word for extended family

2 a traditional Māori tribal meeting place
