# Supplementary material for: The effectiveness of a primary care nursing-led dietary intervention for prediabetes: a mixed methods pilot study
Source: BMC Fam Pract. 2017 Dec 21;18:106. doi: 10.1186/s12875-017-0671-8 (PMC5740796; doi:10.1186/s12875-017-0671-8)
Supplement: Additional file 1: — Prediabetes Intervention Package (PIP) in primary care study process evaluation - key informant and intervention patient interview guides. This file contains copies of the process evaluation interview guide for key informants and the interview guide for intervention participants. (PDF 89 kb) [file 12875_2017_671_MOESM1_ESM.pdf]

Additional file 1: Prediabetes Intervention Package (PIP) in primary care study process evaluation - key informant and intervention patient interview guides.

Key Informants

Preamble

The PIP in primary care study compares the effect of a new prediabetes lifestyle intervention package with usual lifestyle support that is currently offered in general practices. The aim of this qualitative evaluation is to find out whether the intervention package was implemented as intended and to describe what was helpful and what was difficult about implementing dietary changes for patients with prediabetes.

The following areas will be explored with open ended questions:

- Description of your role in the PIP study.
- Your general impressions of how the study implementation process has been.

Probes:

  - Explanation of expectations of their role
  - Information flow
  - Communication
  - Referral processes
  - Relationships between professionals
  - Support given in your role
  - Data collection
  - Patient study information and consent process

- Your observations of which parts of the study implementation process have worked well and why.
- Your observations of which parts of the dietary intervention implementation process have worked well and why.
- Your observations of key areas of challenge for study implementation and why.
- Your observations of key areas of challenge for dietary intervention implementation and why.
- Your suggestions for how these challenges might be best overcome.
- Other recommendations you have for improving the study implementation process.
- Other recommendations you have for improving the dietary intervention implementation process.
- Other comments.

Intervention Patients

Preamble

Thank you for agreeing to take part in this interview, as part of the PIP in primary care study. We are very keen to hear about your experiences and get your feedback.

The following areas will be explored with open ended questions:

- How you felt when you were informed you had prediabetes
- Whether the dietary intervention made a positive difference to the management of your pre-diabetes
- Your experience with the study primary healthcare team - was your particular situation and your pre-diabetes well understood?
- Your observations of the dietary advice given by the study primary healthcare team - was it easily understood? were goals achievable? were phone call reminders useful?
- Your observations of the dietary advice given by the Sports Hawkes Bay team – did you attend? if not, why not? was the course useful? was it easily understood? were phone call reminders useful? was being part of a group with other people with prediabetes helpful?
- What helped you make dietary changes
- What difficulties/barriers did you face when trying to make dietary changes.
- Whether you managed to overcome these difficulties/barriers. If so, how. What parts of the intervention you have managed to maintain and what assisted this.
- What would have made it easier to sustain any dietary improvements.
- Any other comments.
